# Ligation of patent ductus arteriosus in low birth weight premature infants: timing for intervention and effectiveness of bed-side surgery

**DOI:** 10.1186/1749-8090-7-129

**Published:** 2012-12-12

**Authors:** Kıvanç Metin, Fikret Maltepe, Mustafa Kır, Çağatay Bilen, Aslıhan Sökmen, Öztekin Oto, Baran Uğurlu

**Affiliations:** 1 Department of Cardiovascular Surgery, Dokuz Eylül University Faculty of Medicine, 35340, Izmir, Turkey; 2 Department of Anesthesiology, Dokuz Eylül University Faculty of Medicine, Izmir, Turkey; 3 Department of Pediatric Cardiology, Dokuz Eylül University Faculty of Medicine, Izmir, Turkey

**Keywords:** Patent ductus arteriosus, Premature, Surgery, Intensive care unit

## Abstract

**Background:**

Patent ductus arteriosus is a common congenital cardiac condition. Its importance is mostly underestimated and accepted as an “easy” heart disease. Physiological consequences of pulmonary overflow may cause severe mortality in premature neonates. Accurate timing of surgical intervention is essential to decrease the mortality in very low birth weight premature infants. On-site surgery in the intensive care units (ICUs) results excellent surgical quality without jeopardizing the safety of the patients.

**Methods:**

We have summarized the clinical and operative data of 26 premature neonates (<37 weeks of gestational age), which were operated for the diagnosis of PDA in the ICUs of Dokuz Eylül University. Thirteen low birth weight infants (<1000 gr) have been compared with remaining 13 neonates (>1000 gr).

**Results:**

There was no surgical mortality in both groups. Co-existing problems were observed in both groups, which did not affect surgical mortality and morbidity.

**Conclusions:**

Surgery in the ICU is a safe method for premature neonates with physiologically significant PDA. This technique should be the method of choice in experienced centers.

## Background

Patent ductus arteriosus (PDA) is a frequent congenital cardiac condition in low birth weight premature neonates. The consequences of a significant left-to-right shunting through the PDA may present hemodynamic and respiratory importance (chronic lung disease, intraventricular hemorrhage, necrotizing enterocolitis, retinopathy etc.) [1,2]. Spontaneous closure of the PDA in normal birth weight neonates occurs in 3 days, but it may persist longer in prematures [3]. Prolonged endotracheal intubation, mechanical respiratory support and related problems may complicate the condition. Positive effects of early closure of shunting on cardiac and respiratory functions have been reported previously [4]. Systemic treatment options are the method of choice in many centers: Fluid restriction, continuous positive airway pressure and positive end-expiratory pressure ventilator assistance (CPAP + PEEP) and infusion of cyclooxygenase inhibitors (indomethacin and ibuprofen) [5]. Surgical closure is an option for cases where above mentioned techniques may not be sufficient. Timing of surgery is still a controversial issue.

Follow-up conditions of low-birth weight premature neonates are different from other patients. Poor ability of thermal regulation, difficulties for creating access routes (intravenous or nasogastric lines) and challenging endotracheal intubation are limiting factors for those cases, where transportation of prematures from the ICU to the OR is a risky business. Bed-side surgery in the intensive care unit (ICU) conditions is a well described method for capable centers. We have reported bed side PDA ligations in Dokuz Eylül University ICUs between January 1st, 2006 and December 31st, 2011 in this paper.

## Methods

We have retrospectively analyzed 26 surgically intervened PDA cases in Dokuz Eylül University intensive care units (neonatal ICU and cardiovascular surgery ICU) between January 1st, 2006 and December 31st, 2011. Premature infants (<37 weeks of gestational age) without concomitant major cardiac anomalies and persistent ductal shunting despite repeated medical attempts with cyclooxygenase inhibitors are included in this study. All cases have received at least two cures of systemic treatment with cyclooxygenase inhibitors. Transthoracic echocardiography examination was performed in all cases both pre- and postoperatively.

**Figure 1:**
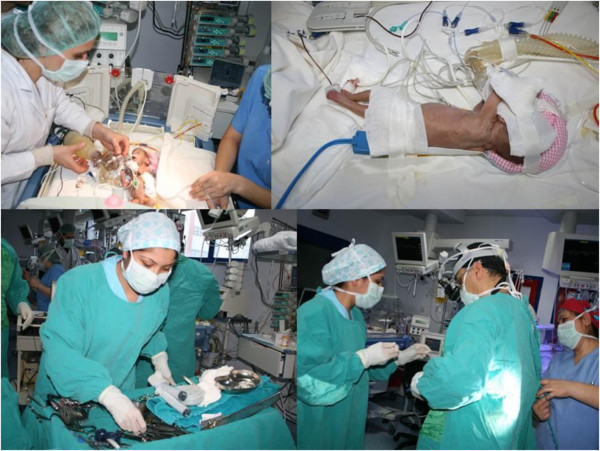
Preparation of the surgical field.

A “bed-side surgery” team, including cardiovascular surgeons, specialists of neonatology and pediatric anesthesiology, scrub nurses and other assisting personnel, has performed the bedside interventions. Appropriate conditions for surgery has been created and controlled by the scrub nurse. Basic surgical armamentarium (instruments, lights, sutures, etc) have been kept in the ICU (Figure 1).

**Figure 2:**
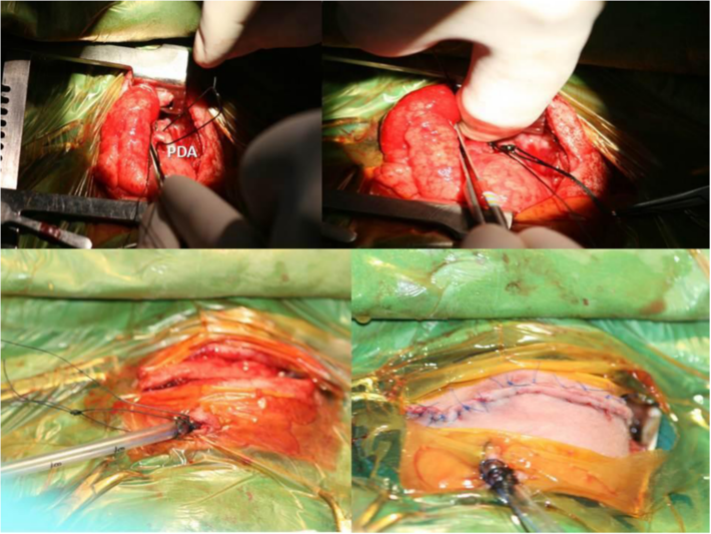
Bed-side double ligation of PDA.

Main reason of bed-side surgery is poor thermal regulation of prematures. All surgical interventions have been performed in the incubator. Simplification of devices and lines with fully monitorization is essential. A fan heater is also an important part of surgical field. All cases were intubated before surgery. Safe lines for medications were created for anesthesiologist and neonatologists. A head light is preferable in addition to mobile light sources. The iodine solution for skin cleaning was preheated to 37°C before surgery. Special surgical instruments in appropriate sizes were chosen. Placement of the electrocautery plaque is a very important issue. Avoiding of getting wet and adequate sizing is essential, where a burn may be extremely dangerous after surgery. A limited standard left posterolateral thoracotomy incision was chosen in all cases. Extrapleural approach to the thorax may be preferable to protect respiratory dynamics, which cannot always be achieved due to fragility of neonatal tissues in cases below 1000 gr. PDAs were doubly ligated and transfixed in standard manner (Figure 2).

Patients were divided in two groups: Group A (<1000 gr of birth weight) and group B (>1000 gram of birth weight). Mothers’ age, gestational age, birth weight, age and weight at the time of the operation, coexisting morbidities, duration of ICU stay and complications (including mortality) were compared between two groups.

All cases were clinically investigated by the pediatric cardiologists and neonatologists both pre- and postoperatively. Infusion of cyclooxygenase inhibitors was the first choice treatment in all cases. Symptomatic cases with persistent shunting after medical attempts were surgically intervened.

The local ethics committee of Dokuz Eylül University has approved for performing this retrospective study on February 16^th^, 2012 (#2012/05-09). Written informed consent was obtained from the parents or relatives of all patients for publication of medical reports and any accompanying images for medical and educational purposes.

Statistically analysis of both groups was made with SPSS for Windows software. Difference between paired samples was calculated with Student’s *T*-test. P < 0,05 was defined as statistically significance.

## Results

Birth weight, gestational age and body weight at the time of operation were significantly different between two groups (p < 0,05) (Table 1).

**Table 1:** Gestational age, birth weight and body weight at the time of operation

**Gestational age (days)**		
**Group A**	222,53 ± 25,7	P = 0,000
**Group B**	179,46 ± 3,8	
**Birth weight (gram)**		
**Group A**	2086,92 ± 876,2	P = 0,000
**Group B**	776,92 ± 252,9	
**Body weight at the time operation (gram)**		
**Group A**	1751,66 ± 727,9	P = 0,019
**Group B**	413,66 ± 667,6	

Mothers’ age, postpartum day of the operation, day of hospital discharge and number of pregnancies were not significantly different between two groups (Table 2).

**Table 2:** Maternal age, postpartum day of operation, day of discharge and number of previous pregnancies

**Mothers’ age (year)**		
**Group A**	31,11 ± 5,9	p > 0,05
**Group B**	26,89 ± 13,2	
**Postpartum day of operation (day)**		
**Group A**	16 ± 12	p > 0,05
**Group B**	23,17 ± 15,7	
**Day of hospital discharge (day)**		
**Group A**	62,7 ± 45,2	p > 0,05
**Group B**	83 ± 33,4	
**Number of pregnancies**		
**Group 1**	2,14 ± 2,2	p > 0,05
**Group 2**	1,29 ± 0,5	

There was no operative and hospital mortality in both groups. The co-existing clinical conditions are summarized in Table 3.

**Table 3:** Coexisting clinical conditions

**Co-existing clinical condition**	**<1000 gr**	**>1000 gr**
**Epilepsy**	1/13	-
**Pneumonia**	3/13	1/13
**Ophthalmic pathology**	1/13	1/13
**Thrombocytopenia**	3/13	-
**Neutropenia**	1/13	-
**Hydronephrosis**	1/13	-
**Congenital hypothyroidism**	2/13	-
**Hydrocephalus**	1/13	-
**Bronchopulmonary dysplasia**	1/13	
**Congenital diaphragmatic hernia**	-	1/13
**Intracranial hemorrhage**	2/13	2/13
**Heart failure**	5/13	4/13
**Pneumothorax**	1/13	1/13
**Paralytic ileus**	1/13	-
**Acute renal failure**	1/13	1/13
**Sepsis**	1/13	1/13
**Omphalitis**	1/13	1/13
**Melena**	1/13	-
**Necrotizing enterocolitis**	1/13	-
**Candida infection**	-	1/13
**Dandy Walker Syndrome**	-	1/13

## Discussion

Functional closure of the *ductus Botalli* occurs in several hours after birth, where anatomical closure may appear in days or weeks [1,6,7]. Functionally closure of the ductus during the first few hours after birth is a desirable event [8]. A surprising reopening of the ductus in premature infants (<30 weeks) results in an increased blood flow through the lungs (left-to-right shunting) and some neonatologists believe that this situation is beneficial for the neonate, but there is no consensus on this topic [8]. PDA is an essential source of mixing in cases with some complex congenital cardiac anomalies and patency of the duct may be the goal of the treatment. On the other hand, significant shunting in otherwise healthy premature infants (<30 weeks of gestational age) may result in hemodynamic and systemic complications [8]. Major complications such as necrotizing enterocolitis and chronic lung disease are important causes of morbidities and have up to 20% mortality rate [1]. Respiratory distress is a consequence of pulmonary immaturity as well as increased pulmonary blood flow. Ligation of the PDA does not guarantee early extubation and ICU discharge. Surgical intervention with thoracotomy incision itself is another factor for respiratory distress. Compression of the left lung may cause parenchymal bleeding and postoperative atelectasis. Surgically intervened cases receive several attempts of medical treatments and during that period, some complications occur, which are affecting the mortality rate in those patients [1]. Repeated attempts for medical closure of PDA have been shown to increase the risk of concomitant surgery [1,7].

The incidental discovery of the effects cyclooxygenase inhibitors on *ductus Botalli* has opened the era of medical treatment for persistently PDA [2]. Treatment of PDA is a classic but still controversial issue. It’s clear that not all cases with PDA are candidates for surgery. Important questions are the dosage, duration and number of medical treatments and timing for the surgery [1,8]. The policy for closing all significant PDAs is a matter of discussion. The criteria are uncertain and timing is controversial: To close or not to close? That’s the question! [8].

Persistency of the ductus after 3 postpartum days in termed neonates is accepted as a pathological condition. But spontaneous closure delays in premature neonates and most of the neonatologists accept this situation as a “normal” physiological clinical status. They conclude that surgical or medical closure of the PDA in preterm infants may negatively interfere with postnatal adaptation. An opposite opinion is that prolonged patency of the duct may result in severe side effects, and therefore it has to be closed [4]. Displacement of the blood flow to the pulmonary vasculature rather than abdominal organs results in impaired intestinal perfusion, malnutrition and necrotizing enterocolitis [5,9]. Early surgical closure shortens the delay of oral nutrition in neonates and improves body growth [3]. In addition, early closure of the PDA regulates pulmonary blood flow and decreases pulmonary edema, the risk of acute respiratory complications and necessity of mechanical ventilation. Bronchopulmonary dysplasia is an important consequence and has to be concluded beyond the acute respiratory problems. It is affected by inhaled high oxygen concentration in the first 4 hours of age [3]. Timing of the surgery, i.e. before the onset of the respiratory symptoms, is the main determinant. PDA related mortality can be far more than expected. It has been reported up to 20%! [1]. Bad timing for surgery does not reduce this rate [10]. Important question is “how many attempts of medical treatment” should be tried before surgical intervention? Surgery has to be the first method in unstable cases with pulmonary edema and heart failure. Medical treatment attempts may result in losing of very valuable time and increase operative mortality. Early ligation of PDA significantly reduces the duration of mechanical ventilation and its complications [3]. Symptomatic patients with PDA have to be intervened surgically; even there is no sign of heart failure and pulmonary edema. Early surgical closure have very low mortality and 4% to 10% morbidity [3,7].

Larger, older (as gestational age and time of surgery) neonates do require shorter duration of mechanical ventilation and better survival rates [1]. Although there is no cut-off point to decide the timing of intervention, there are no absolute contraindications for PDA closure in small, premature, and preoperatively unstable patients [1]. Another data supporting the advantages of surgical intervention in premature neonates was reported from a center without readily accessible pediatric cardiac surgery team. Mortality was three times higher in cases with persistent ductal shunting after 2 courses of medical attempts compared to patients, whose PDAs closed with indomethacin treatment (33% vs 11%) [1,7]. Surgical ligation was superior to medical treatment in a larger series of very-low-birth-weight neonates [1,11].

What is the goal of “bed-side” surgery? This is another important issue of this topic. Low birth weight premature infants are under risk of hypothermia and dislodgement of vascular lines during transportation. Modern ICUs are facilities with improved technical capabilities including surgical interventions. Many surgical procedures (such as revision surgery, embolectomy, chest closure after open sternum operations etc.) are routinely performed in experienced cardiac ICUs. Although surgical interventions are not common in neonatal ICUs, an appropriately designed and dedicated team, which has experience of surgery in the ICUs, can perform effective operations in this environment. We are performing surgery for very low birth weight infants in our ICUs, including the neonatal ICU.

## Conclusion

The aim of our study is to prove the efficacy of bedside surgical intervention in the ICU, which is preferable for premature neonates. We have concluded that the risk of transportation in those cases could be overcome with this technique without jeopardizing the safety of the patient.

## Abbreviations

PDA: Patent Ductus Arteriosus; CPAP: Continuous Positive Airway Pressure; PEEP: Positive End-Expiratory Pressure.

## Competing interests

Hereby we declare that there are no competing interests of any above mentioned co-authors regarding to preparation of this paper.

## Authors’ contributions

Assoc. Prof. KM, Prof. ÖO and Prof. BU are surgeons performing most of the operations. Dr. ÇB is the resident who has collected the data. Ass. Prof. MK is the attending pediatric cardiologists performing the diagnostic tests. Assoc. Prof. FM is the responsible anesthesiologist. AS is the scrub nurse. All co-authors have intellectually supported the study. KM is the corresponding author who has written the paper. All authors read and approved the final manuscript.
